# Water pressure–assisted endoscopic stricturotomy with band-assisted mucosectomy and polypectomy for a complex colonic stricture in Crohn’s disease guided by preprocedure intestinal ultrasound

**DOI:** 10.1016/j.igie.2025.09.004

**Published:** 2025-09-05

**Authors:** Partha Pal, Mohammad Abdul Mateen, Pradeep Rebala, Rajesh Gupta, Manu Tandan, D.Nageshwar Reddy

**Affiliations:** 1 Medical Gastroenterology, Asian Institute of Gastroenterology, Hyderabad, India; 2 Diagnostic Radiology and Imaging, Asian Institute of Gastroenterology, Hyderabad, India; 3 Surgical Gastroenterology, Asian Institute of Gastroenterology, Hyderabad, India

A 39-year-old man with colonic Crohn’s disease for 4 years for which he was taking azathioprine and mesalamine presented with progressive obstructive symptoms for 18 months. He underwent endoscopic balloon dilation (12 mm) for a sigmoid-descending junction stricture without symptom relief 9 months previously. Ultrasound scan of the intestines revealed a short, fibrotic stricture with prestenotic dilation at the sigmoid-descending junction (Fig. 1A).

**Figure 1:**
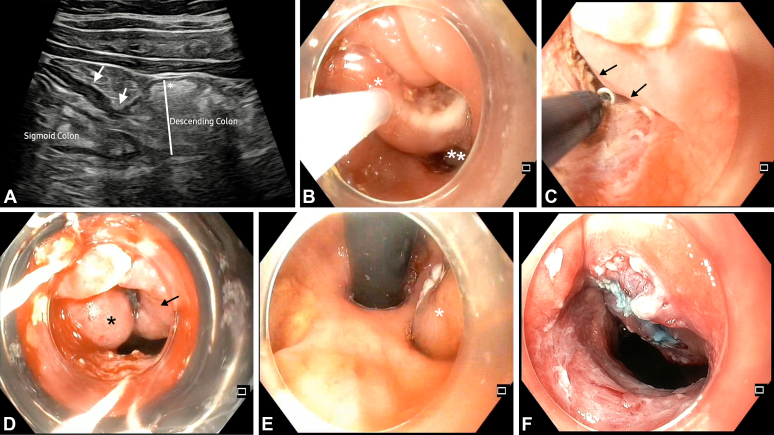
**A,** Transabdominal ultrasound showing a short-segment fibrotic stricture (*arrows*) at the sigmoid-descending junction with prestenotic dilation (*asterisk*). **B,** Pseudopolyps distal (*single asterisk*) and proximal (*2 asterisks*) to the stricture. Piecemeal snare polypectomy being performed for the proximal pseudopolyps. **C,** Water pressure–assisted endoscopic stricturotomy being performed using an insulated-tip knife; water jet displaces opposing mucosa (*arrows*) to avoid thermal injury. **D,** Band ligation performed for protruding folds proximal to the stricture showing inadequate capture: *asterisk* showing captured fold and *arrow* showing part of the obscuring fold that is not captured by the band in between. This led to paradoxical worsening as it blocked the stricture opening. **E,** Retroflexed view from the descending colon poststricturotomy showing obstructing mucosal folds (*asterisk*) requiring mucosectomy. **F,** Poststricturotomy, polypectomy, and mucosectomy image with adequate luminal opening.

Colonoscopy confirmed the stricture, complicated by multiple pseudopolyps obscuring the lumen (Fig. 1B, *asterisks*). Initial polypectomy was performed to clear the visual field, followed by endoscopic stricturotomy (ES) using an insulated-tip knife with electrocautery (ENDO CUT I, settings 3-1-3; Erbe Elektromedizin GmbH, Tübingen, Germany). However, luminal orientation remained challenging because of adjacent mucosal folds and pseudopolyps protruding from the proximal segment.

To manage this, we adopted a water pressure–assisted ES technique: intermittent water-jet irrigation was used to gently distend and displace the opposite mucosal folds away from the ES plane (Fig. 1C). This allowed a safer and more-controlled radial and circumferential incision of the stricture without contact with the opposing mucosa.

Poststricturotomy, the pediatric colonoscope could be advanced across the stricture, but luminal narrowing persisted as the result of bulky overhanging proximal mucosa. An attempt at band ligation of the obstructing folds paradoxically worsened the obstruction because of inadequate capture (Fig. 1D). Targeted mucosectomy of the remaining obstructive pseudopolyps was performed after submucosal injection. The mucosa beneath the previous band was resected, and further excision of obstructive folds was achieved under direct vision (Video 1, available online at www.igiejournal.org). The study was approved by the institutional ethics committee (Asian Institute of Gastroenterology/Institutional Ethics Committee – Basic Health and Research 32/07.2022-05).

Retroflexion proximal to the stricture helped decide the extent of mucosectomy (Fig. 1E). After adequate mucosectomy led to satisfactory luminal diameter (Fig. 1F), 2 hemostatic clips were applied at the mucosectomy site to prevent delayed bleeding and perforation. The patient reported substantial relief of abdominal discomfort and constipation. Budesonide-multimatrix tablets were started for their local anti-inflammatory effect, with emerging evidence of benefit in preventing postendoscopic submucosal dissection colonic strictures, although their role in fibrosis prevention remains unproven.^1^ There was no active inflammation elsewhere. Endoscopic biopsies ruled out dysplasia. At 3-month follow-up, the patient remained clinically asymptomatic.

The water-pressure method has been shown to improve safety and visualization during endoscopic submucosal dissection by creating a more-controlled dissection plane.^2^ We extrapolated this principle to ES in a complex Crohn’s disease stricture. To our knowledge, this is the first case demonstrating a novel adaptation of water-pressure technique in ES in a Crohn’s disease stricture complicated by pseudopolyps and protruding mucosa guided by intestinal ultrasound.^3^ It enabled safe dissection, enhanced visualization, and facilitated effective stricture resolution in a challenging anatomical setting. Nonetheless, this technique should be used with caution, especially in fibrotic strictures, given the risk of perforation (pooled perforation rate ∼2.4%).^3^

## Patient Consent

Written informed consent was taken from the patient for the publication of the information and imaging.

## Disclosure

The following author disclosed financial relationships: P. Pal: Consultant for Johnson & Johnson. All other authors disclosed no financial relationships.

## Supplementary data

Video 1 Step-by-step demonstration of water pressure-assisted endoscopic stricturotomy, polypectomy, and band-assisted mucosectomy for a complex descending colon stricture in Crohn disease. Includes insulated-tip knife-assisted stricturotomy, use of water jet to guide incision, mucosectomy and polypectomy, and final luminal result including placement of hemoclips.
